# The impact of environmental acidification on the microstructure and mechanical integrity of marine invertebrate skeletons

**DOI:** 10.1093/conphys/coz062

**Published:** 2019-11-11

**Authors:** Maria Byrne, Susan Fitzer

**Affiliations:** 1 School of Medical Science and School of Life and Environmental Science, The University of Sydney, NSW 2006, Australia; 2 Institute of Aquaculture, University of Stirling, Stirling, FK9 4LA, UK

**Keywords:** Climate change, marine biominerals, corals, molluscs, sea urchins, serpulid worms

## Abstract

Ocean acidification (OA), from seawater uptake of anthropogenic CO_2,_ has a suite of negative effects on the ability of marine invertebrates to produce and maintain their skeletons. Increased organism *p*CO_2_ causes hypercapnia, an energetically costly physiological stress. OA alters seawater carbonate chemistry, limiting the carbonate available to form the calcium carbonate (CaCO_3_) minerals used to build skeletons. The reduced saturation state of CaCO_3_ also causes corrosion of CaCO_3_ structures. Global change is also accelerating coastal acidification driven by land-run off (e.g. acid soil leachates, tannic acid). Building and maintaining marine biomaterials in the face of changing climate will depend on the balance between calcification and dissolution. Overall, in response to environmental acidification, many calcifiers produce less biomineral and so have smaller body size. Studies of skeleton development in echinoderms and molluscs across life stages show the stunting effect of OA. For corals, linear extension may be maintained, but at the expense of less dense biomineral. Conventional metrics used to quantify growth and calcification need to be augmented by characterisation of the changes to biomineral structure and mechanical integrity caused by environmental acidification. Scanning electron microscopy and microcomputed tomography of corals, tube worms and sea urchins exposed to experimental (laboratory) and natural (vents, coastal run off) acidification show a less dense biomineral with greater porosity and a larger void space. For bivalves, CaCO_3_ crystal deposition is more chaotic in response to both ocean and coastal acidification. Biomechanics tests reveal that these changes result in weaker, more fragile skeletons, compromising their vital protective roles. Vulnerabilities differ among taxa and depend on acidification level. Climate warming has the potential to ameliorate some of the negative effects of acidification but may also make matters worse. The integrative morphology-ecomechanics approach is key to understanding how marine biominerals will perform in the face of changing climate.

## Introduction

Ocean acidification (OA) resulting from increased ocean uptake of CO_2_, driven by the increase in anthropogenic greenhouse gas emissions, is unprecedented on geological time scales (Zeebe *et al*., 2016). This uptake is causing major changes to ocean chemistry with a suite of negative effects on the ability of marine invertebrates to produce and maintain their skeletons. Firstly, increased organism *p*CO_2_ causes hypercapnia, a stress that can affect many physiological processes. Hypercapnia can be energetically costly reducing the resources that animals need to calcify because maintenance of essential metabolic processes (e.g. acid-base homeostasis) takes priority over diverting energy to growth and calcification (Stumpp *et al*., 2012; Pan *et al*., 2015). The production and maintenance of calcium carbonate (CaCO_3_) structures has been shown to be modulated by energy acquisition (Melzner *et al*., 2011). Secondly, OA reduces the saturation state of CaCO_3_ minerals, limiting the carbonate available for calcification (IPCC, 2014). These minerals are the building blocks of biocalcification and include aragonite and calcite. Thirdly, the direct corrosive effect of OA can cause pitting and erosion of CaCO_3_ structures, as reported for calcifiers that are resident in low pH CO_2_ seep environments (Harvey *et al*., 2018). Calcite is less susceptible to dissolution at lower pH values than aragonite, unless it contains high levels of magnesium (Ries *et al*., 2009; Chan *et al*., 2012). However, calcite is more brittle compared to aragonite, making it mechanically weaker (Fitzer *et al*., 2015a; Meng *et al*., 2018).

**Figure 1:**
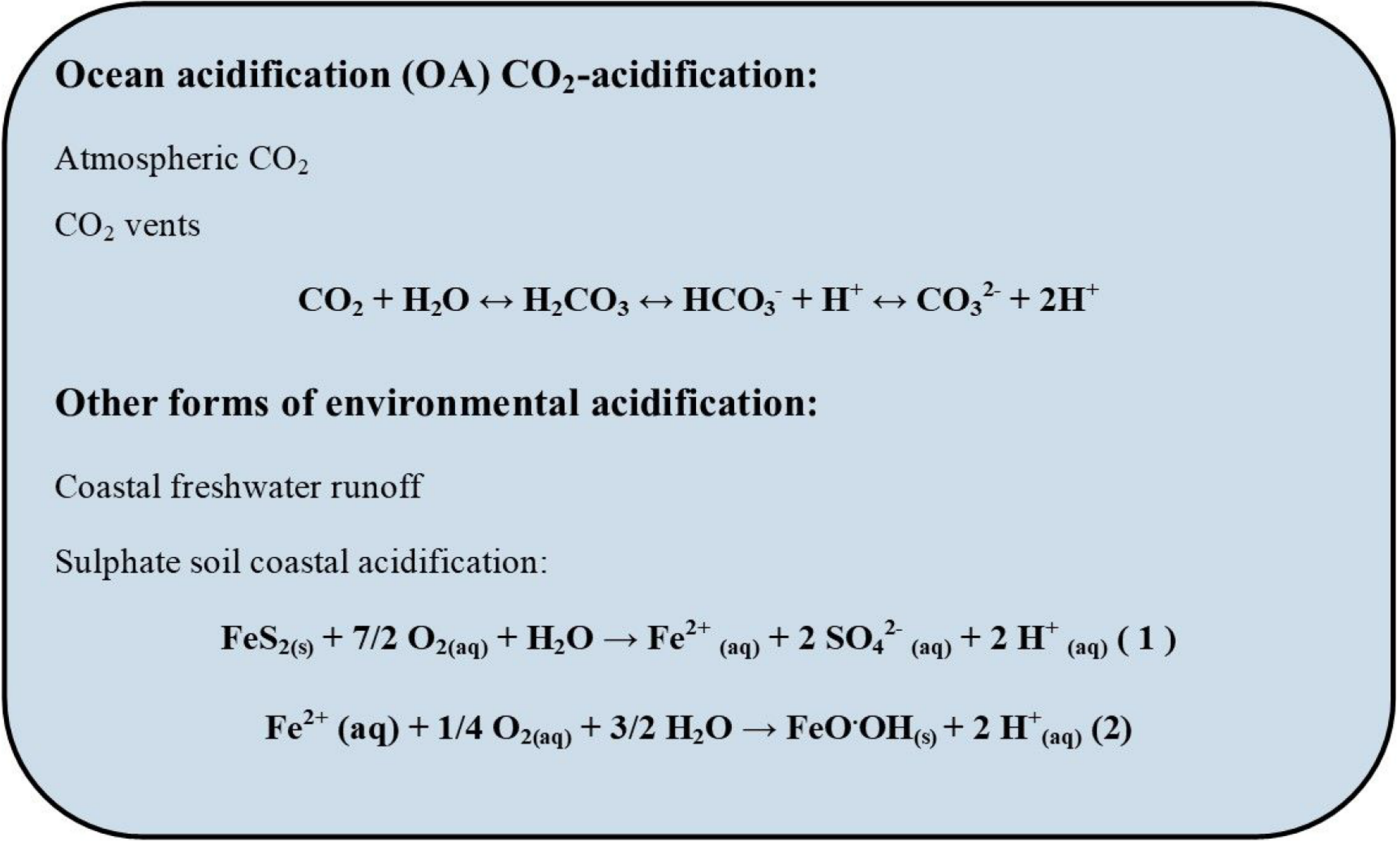
Equations for the mechanisms of acidification from CO_2_-OA and other forms of environmental acidification for example from acid sulphate soil leachates. Modified from Fitzer *et al*. (2018).

Due to sea-level rise and changing weather patterns, global change is exacerbating coastal acidification in areas where freshwater runoff results in reduced pH due to leachate from acid sulphate soils and humic acids and tannic acids from groundwater (Amaral *et al*., 2011, 2012; Duarte *et al*., 2013; Jiang *et al*., 2017; Fitzer *et al*., 2018). Environmental acidification in a changing ocean is being caused by both ocean and coastal acidification. These two forms of acidification (CO_2_ and land run off) differ in chemistry mechanisms (Fig. 1). Environmental acidification also occurs at CO_2_ seeps (Foo *et al.* 2018; González-Delago and Hernández, 2018).

Marine invertebrate skeletons are complex and ancient structures (Wilkinson, 1979) produced through the superb biocontrol of mineral production, resulting in a great diversity of taxon-specific structures (Wilbur, 1972; Cavey and Markel, 1994; Von Euw *et al*., 2017). For a broad range of marine invertebrates (e.g. corals, molluscs, echinoderms) experimental acidification in laboratory studies and environmental acidification at natural CO_2_ seeps and in low pH coastal waters (Amaral *et al*., 2011, 2012; Duarte *et al*., 2013; Jiang *et al*., 2017; Fitzer *et al*., 2018) show the negative effect of acidification on biomineralisation. Faced with these challenges, building and maintaining calcified structures by marine invertebrates will depend on the balance between calcification and dissolution.

Calcified structures are expensive to produce (Comeau *et al*., 2017) and, in response to the physiological challenges presented by OA, many calcifiers produce smaller skeletons and so have smaller body sizes. This is seen in the stunting effect of OA at the larval and adult stage of molluscs and echinoderms (Parker *et al*., 2012, 2013; Byrne *et al*., 2013; Garilli *et al*., 2015; Dworjanyn and Byrne, 2018). Vulnerabilities with respect to the amount of biomineral produced, its mechanical properties and propensity for dissolution or etching in OA conditions differ greatly among taxa, even within the same phylum that have similar calcification mechanisms (Table 1). Most laboratory studies have involved translocation of animals that that have calcified (i.e. ‘grown up’) in control conditions to OA conditions for short- or long-term exposure, an approach that shows the effects of OA on established skeleton and the ability to make new skeleton. For insight into the inherent differences of the production of biomineral in normal ambient and acidification conditions, many studies have reared larvae and juveniles under OA (Parker *et al*., 2012, 2013; Byrne *et al*., 2013; Dworjanyn and Byrne, 2018) or have availed of naturally low pH habitats (e.g. CO_2_ seeps or low pH coastal waters) where biomineralisation has occurred through life (Crook *et al*., 2013; Garilli *et al*., 2015; Fitzer *et al*., 2018; Foo *et al*., 2018; González-Delago and Hernández, 2018; Migliaccio *et al*., 2019).

**Table 1:** Studies that have investigated the impacts of environmental acidification on the microstrucure and/or mechanics of marine invertebrate skeletons and methods used. pH levels are listed as published on the total (pHT) or NBS (pHNBS) scales or no scale (pH*) if not indicated

Phylum/ species	Time	pH or Ω levels/location	Morph methods	Morph results	Biomechanics methods	Biomechanics results	References
**Cnidaria**							
*Balanophyllia* *europaea*	Resident	pHT 7.7, 7.9, 8.1; Vent	μCT, SEM	pHT 7.7, 21-31% ↑ in porosity & 7% ↓ in bulk density	Nanoindentation	pHT 7.7, hardness - no effect of pH; ↓ in stiffness	Fantazzini *et al*., (2015)
*Favia fragum*	8 d to juvenile**	Ω Ar 0.22-3.71; Lab	SEM	Ω Ar ≤ 1; change in crystal size, shape & orientation, thin septa	-	-	Cohen *et al*., (2009)
*Porites astreoides*	Resident	Ω Ar < 1-3.71; Coast	μCT	Ar < 2; ↓ density	-	-	Crook *et al*., (2013)
*Stylophora pistillata*	12 mo	pHT 7.2, 7.4, 7.8, 8.0; Lab	μCT	pHT 7.2-7.4, ↑ in porosity & ↓ in bulk density, thinner skeleton, no change in crystallography	-	-	Tambutté *et al*., (2015)
*Stylophora pistillata*	14 mo	pH _NBS_ 7.3, 7.6, 8.2; Lab	SEM, EBSD	pH _NBS_ 7.3, 7.6, crystallographic changes, shorter less round fibre bundles	-	-	Coronado *et al*., (2019)
**Brachiopoda**							
*Magellania venosa*	335 d	pH* 7.35-8.15 Lab, ASW	SEM	pH* 7.35-7.6, ↑ pore size; ↑ thickness primary layer; ↓ thickness secondary layer, more organic rich shell	-	-	Ye *et al*., (2018)
**Annelida**							
*Hydroides elegans*	9-18 d**	pH_NBS_ 7.4, 7.6, 7.8,7.9, 8.0; Lab	μCT, SEM	pH_NBS_ 7.4-7.8, ↑ porosity, irregular layers, surface pitting & erosion, thinner; change in mineral layers & crystallography	Nanoindentation, crushing test	pH_NBS_ 7.4-7.8, ↓ hardness, elasticity & 62% ↓ in crushing force	Chan *et al*., (2012); Li *et al*., (2014; 2016)
**Mollusca**							
**Gastropoda**							
*Austrocochlea constricta*	4 mo	pH_NBS_ 7.8, 8.1; Lab	-	-	Vickers hardness, Elastic modulus	pH_NBS_ 7.8, ↑ shell hardness, ↑ stiffness	Leung *et al*., (2017)
*Austrocochlea odontis*	4 mo	pH_NBS_ 7.86, 8.0; Lab	-	-	Vickers hardness, Elastic modulus	No effect of pH	Leung *et al*., (2017)
*Austrocochlea porcata*	95 d	pH_NBS_ 7.7, 7.9, 8.1; Lab	-	No effect of pH	Crushing test	pH_NBS_ 7.7, ↓ shell strength by 28%	Coleman *et al*., (2014)
*Bulla quoyii*	4 mo	pH_NBS_ 7.9, 8.1; Lab	-	-	Vickers hardness, Elastic modulus	No effect of pH	Leung *et al*., (2017)
*Charonia lampas*	Resident	pHT 7.8, 8.1; Vent	μCT	pHT 7.8, Two-fold ↓ in density and thickness	-	-	Harvey *et al*., (2018)
*Columbella rustica*	3 mo	pH_NBS_ 7.6, 8.1, Lab	μCT	pH_NBS_ 7.6, 0.8-8% ↓ in density depending on shell region	-	-	Chatzinikolaou *et al*., (2017)
*Littorina littorea*	5 mo (small and large)	pH_NBS_ 7.8, 8.0; Lab	-	-	Crushing test	pH_NBS_ 7.8, Large snails, ↓ in crushing force, Small snails, no effect of pH	Landes and Zimmer, (2012)
*Nassarius nitidus*	3 mo	pH_NBS_ 7.6, 8.1, Lab	μCT	pH_NBS_ 7.6, 38-51% ↓ in density depending on shell region	-	-	Chatzinikolaou *et al*., (2017)
*Nerita atramentosa*	4 mo	pH_NBS_ 7.8, 8.1; Lab	-	-	Vickers hardness, Elastic modulus	No effect of pH	Leung *et al*., (2017)
*Nucella lapillus*	14 mo (juveniles)	pHT 7.8, 7.9, 8.0; Lab	μCT, SEM	pHT 7.8, 20-30% ↓ in density; surface erosion, lack of layering in shell, younger shells tinner, older shells thicker	-	-	Queirós *et al*., (2015); Rühl *et al*., (2017)
*Nucella ostrina*	6 mo.	pHT ~ 7.6, 8.1; Lab	-	-	Crushing test	10% reduction in strength	Barclay *et al.,* (2019)
*Phasianella australis*	4 mo	pH_NBS_ 7.9, 8.1; Lab	-	-	Vickers hardness, Elastic modulus	No effect of pH	Leung *et al*., (2017)
*Phorcus sauciatus*	Resident	pHNBS 7.52-8.2 Vent	-	Vent site – corrosion and cracks in shell	Crushing test	Weaker shells	Viotti *et al*., (2019)
*Subninella undulata*	65 d	pH_NBS_ 7.7, 7.9, 8.1; Lab	-	pH_NBS_ 7.7, ↓ shell thickness	Crushing test	No effect of pH	Coleman *et al*., (2014)
*Tegula funebralis*	6 mo.	pHT 7.4-8.1; Lab	-	-	Crushing test	50% reduction in strength	Barclay *et al.,* (2019)
*Thalotia conica*	4 mo	pH_NBS_ 7.9, 8.1; Lab	-	-	Vickers hardness, Elastic modulus	No effect of pH	Leung *et al*., (2017)
*Turbo undulatus*	4 mo	pH_NBS_ 7.86, 8.0; Lab	-	-	Vickers hardness, Elastic modulus	No effect of pH	Leung *et al*., (2017)
**Bivalvia**							
*Adamussium colbecki*	1 mo	pHT 7.6, 8.1; Lab	SEM	No effect on crystals	Nanoindentation	No effect of pH	Dell’Acqua et al., (2019)
*Arctica islandica*	3 mo	pH_NBS_7.7, 7.9, 8.0	SEM	No effect on shape or size of crystals	-	-	Stemmer *et al*., (2013)
*Cerastoderma edule*	2 mo	pH_NBS_, 6.4 6.7, 7.0, 7.4, 7.8, Lab	SEM	pH_NBS_ 6.4, 6.7 ↓ net calcification, no change in dissolution	Nanoindentation	No effect of pH	Milano *et al*., (2016)
*Crassostrea virginica*	20 wk (juveniles) 2 wk (adults)	pH_NBS_ 7.5, 8.2, Lab	FTIR SEM	pH_NBS_ 7.5 40% ↓ in shell mass in juveniles	Microindentation, fracture toughness	pH_NBS_ 7.5 ↓ calcite hardness and fracture toughness in juveniles	Beniash *et al*., (2010)
*Crassostrea virginica*	11 wk	pH_NBS_ 7.97/8.1 8.11/8.36 Lab	SEM	No difference in shell body mass	Microindentation, fracture toughness	pH_NBS_ 7.9/8.1 ↓ hardness and fracture resistance at salinity	Dickinson *et al*., (2012)
*Magallana angulata*	35 d early juveniles	pH_NBS_ 7.8, 8.1, Lab	SEM, EBSD μCT,	pH_NBS_ 7.8 ↑ porosity of microstructure, ↓ density	Nanoindentation	pH_NBS_ 7.8 ↓ hardness and stiffness	Meng *et al*., (2018)
*Magallana gigas*	6 wk	pH_NBS_, 7.7, 8.1, Lab	-	-	Crushing test	pH_NBS_ 7.7 ↓ crushing force	Wright *et al*., (2018)
*Mytilus californianus*	8 d larva**	pH_NBS_ 7.8, 8.0, 8.1, Lab	SEM	pH_NBS_ 7.8 15% thinner	Crushing test	pH_NBS_ 7.8 15-20% weaker pHNBS 8.0 13-15% weaker	Gaylord *et al*., (2011)
*Mytilus edulis*	6 mo	pH_NBS_7.2, 7.3, 7.4, 7.5, 7.7, 8.1, 8.2, Lab	SEM, EBSD	pH_NBS_ 7.2, 7.3, 7.4, 7.5. 7.7 disorganised crystals & altered layer structure	-	-	Fitzer *et al*., (2014b)
*Mytilus edulis*	6 mo (juveniles)	pH_NBS_7.2, 7.3, 7.4, 7.5, 7.7, 8.1, 8.2, Lab	SEM, EBSD	pH_NBS_7.2, 7.3, 7.4, 7.5, thinner calcite layer and altered layer structure and crystallography	-	-	Fitzer *et al*., (2014a)
*Mytilus edulis*	6 mo	pHT 7.65, 8.0; Lab	-	-	Crushing test	pHT 7.65 ↓ flex before failure; Strength not affected	Mackenzie *et al*. (2014)
*Mytilus edulis*	6 mo	pH_NBS_7.2, 7.3, 7.4, 7.5, 7.7, 8.1, 8.2, Lab	-	-	Microindentation fracture toughness, nanoindentation	pH_NBS_7.4, 7.5,7.7 ↑ calcite hardness and ↓ fracture toughness	Fitzer *et al*., (2014a)
*Mytilus edulis*	2 mo	pH_NBS_ 7.8, 8.1, Lab	SEM	Disordered crystals	Crushing test	pH_NBS_ 7.8, ↓ crushing strength 22-24%	Li *et al*., (2015)
*Mytilus edulis*	6 mo	pH_NBS_ 7.4, 8.1, Lab	SEM-EBSD, XPEEM	pH_NBS_ 7.2, altered crystallography structure and more ACC.	-	-	Fitzer *et al*., (2016)
*Mytilus edulis*	9 months	pH_NBS_ 7.2, 7.3, 7.4, 7.5, 7.7, 8.1, 8.2, 8.2, 8.1, Lab	Shell shape analysis, shell thickness index (STI)	pH_NBS_ 7.2, 7.3, 7.4, 7.5, 7.7, ↓ shell thickness and rounder flatter shells.	-	-	Fitzer *et al*., (2015b)
*Mytilus edulis*	7 weeks	pH_NBS_7.2, 7.4, 7.7, 8.0, Lab	SEM	pH_NBS_7.2, corrosion of internal aragonite layers	-	-	Melzner *et al*., (2011)
*Mytilus edulis*	6 mo	pHT 7.65, 8.0; Lab	-	-	Crushing test	pHT 7.65 ↓ flex before failure; Strength not affected	Mackenzie *et al*. (2014)
*Mytilus galloprovincialis*	68 d	pHT 7.25, 8.07; Vent transplant	SEM, EBSD	pHT 7.25, thinner shell, disturbed less ordered structure	-	-	Hahn *et al*., (2012)
*Mytilus galloprovincialis*	21 d - 5 mo	pHT 6.8, 7.2, 7.8, Vent transplant to lab	SEM	Calcification continued in vents; Isolated shells, dissolution at low pH	-	-	Rodolfo-Metalpa *et al*., (2011)
*Pinctada fucata*	28 d	pH_NBS_7.6, 7.8, 8.1	SEM	Shell dissolution	Crushing test	pH_NBS_ 7.6, 25.9% and 26.8% weaker shells	Welladsen *et al*., (2011)
*Saccostrea glomerata*	2 yr	pH_NBS_ 7.7, 8.1 Coast***	SEM-EBSD	pH 7.7, disordered crystallographic structure	-	-	Fitzer *et al*., (2018, Fitzer *et al*., 2019b)
*Saccostrea glomerata*	N/A	pH_NBS_ 6.6, 6.8, 6.9, 7.8, 7.9 Coast***	-	-	Crushing test	pH pH_NBS_ 6.6, 6.8, 6.9, Weaker shells	Amaral *et al*., (2012); Fitzer *et al.,* (2019b)
**Cephalopoda**							
*Argonauta nodosa*	N/A	pHT 7.35,7.55,7.8,8.0, Lab	SEM, EBSD	Shell only pHT 7.35-7.8, dissolution and etching, altered crystallography and structure	-	-	Wolfe *et al*., (2012, 2013a)
**Echinodermata**							
*Diadema africanum*	100 d, juveniles	pHT 7.6, 8.0, Lab	SEM	pHT 7.6, Test plates thinner, spines, dissolution/etching	Crushing test (whole urchin dried)	pHT 7.6, ↓ crushing force	Rodríguez *et al*., (2017)
*Echinometra* sp.	343 d	pH_NBS_ 7.7, 8.1, Lab	SEM	Test plates and sines, no dissolution	-	-	Hazan *et al*., (2014)
*Echinometra mathei*	13 mo	pHT 7.65, 8.1, Lab	-	-	Ambital and apical plate fracture force and elasticity	No effect of pH	Moulin *et al*., (2015)
*Heliocidaris erythrogramma*	2 wk, early juveniles	pH_NBS_7.4, 7.6, 7.8, 8.1, Lab	SEM	pH_NBS_ 7.4, Spines, ↑ pore size and dissolution	-	-	Wolfe *et al*., (2013b)
*Heliocidaris erythrogramma*	9 mo	pH_NBS_ 7.6, 8.1, Lab	SEM	pH_NBS_ 7.6, ↑ pore size apical but not ambital plates	Nanoindentation, elasticity	pH_NBS_ 7.6, ↓ hardness and elasticity	Johnson and Byrne (unpublished data)
*Lytechinus variegatus*	3 mo, early juveniles	pH_NBS_ 7.8, 8.0, 8.1, Lab	SEM	pH_NBS_ 7.4, Spines, dissolution and malformation	-	-	Albright *et al*., (2012)
*Lytechinus variegatus*	59 d	pH* 7.47, 7.7, 7.9, Lab	SEM	pH* 7.47, Spines, reduced barbs	Snap test	pH* 7.47, ↓ snap force	Emerson *et al*., (2017)
*Paracentrotus lividus*	1 mo (juveniles)	pHT 7.7, 7.8, 8.0, 8.1, Lab	SEM	pHT 7.7, larger pore size in tooth, no change test plate thickness; no spines dissolution/etching	Crushing test (whole urchin dried) Fracture force plate	pHT 7.7, ↓ crushing force; No effect of pH on test plate	Asnaghi *et al*., (2013), 2014, (2019)
*Paracentrotus lividus*	12 mo	pH_NBS_ 7.8, 7.9, 8.0, 8.1, Lab	-	-	Crushing tests, ambital and apical plate fracture force, nanoindentation, elasticity	No effect of pH	Collard *et al*., (2016)
*Paracentrotus lividus*	Resident	pH_NBS_ 7.8, 8.2, Vent	-	-	Crushing force, ambital and apical plate fracture force, nanoindentation, elasticity	No effect of pH	Collard *et al*., (2016)
*Paracentrotus lividus*	100 d, juveniles	pHT 7.6, 8.0, Lab	SEM	pHT 7.6, Test plates thinner, Spines, no dissolution/etching	Crushing test (whole urchin dried)	pHT 7.6, ↓ crushing force	Rodríguez *et al*., (2017)
*Tripneustes gratilla*	146 d juvenile to adult	pH_NBS_ 7.6, 7.8, 8.1 Lab	SEM	pH_NBS_ 7.6, thinner test, spines no dissolution/etching	Crushing test (live whole urchin)	pH_NBS_ 7.6, ↓ crushing force, rupture at sutures not skeleton	Byrne *et al*., (2014)
*Tripneustes ventricosus*	5 wk	pHT 7.4, 7.7, 8.1, Lab	SEM	pHT 7.4, 7.7, spine etching Test plates, no dissolution/etching	Fracture force spines, two-point bending, elasticity	pHT 7.4, 7.7, spine more brittle, ↓ fracture force, 35% and 16%, no effect of pH on elasticity	Dery *et al*., (2017)
*Strongylocentrotus droebachiensis*	45 d	pH_NBS_ 7.2, 7.7, 8.0, Lab	SEM	pH_NBS_ ~ 7.2, test plates pitted; spine dissolution	Fracture force spines	pH_NBS_ ~ 7.2, spines, ↓ fracture force; No effect of pH on test plates	Holtmann *et al*., (2013)
**Crustaceans**							
*Amphibalanus amphitrite*	86 d-20 wk	pH_NBS_ 7.4, 8.2; Lab	-	-	Penetrometry, breaking test	pH_NBS_ 7.4, ↓ force to penetrate shells	McDonald *et al*., (2009)
*Amphibalanus improvisus*	86d-20 wk	pHT 7.3-7.9 Lab	SEM	Low pH increasing corrosion	Crushing test	No effect of pH	Panch *et al*., (2013, 2014)

Morphology methods: EBSD, electron back scatter defractometry; FTIR, Fourier-transform infrared spectroscopy; μCT, micro-computed tomography; SEM, scanning electron microscopy; XPEEM, photo emission electron microscopy. Biomechanics methods: crushing tests (force of rupture); nanoindentation (hardness; Young’s modulus of elasticity—a measure of stiffness). ACC, amorphous calcium carbonate; ASW, artificial seawater; Coast, ***coastal runoff pH gradient; Lab, laboratory; Morph, morphology; Ω, saturation state calcium carbonate minerals; Time, exposure duration; Vent, CO_2_ seep pH gradient; −, not investigated; **, larva to juvenile studies, all others are of adults; ***, includes pH flux

The impact of OA on calcification depends on level of *p*CO_2_, with near future projections (e.g. pH_T_ 7.8) having milder impacts than far future ones (e.g. pH_T_ 7.6 and lower) and also depends on the duration of exposure (see Table 1). For instance, many calcifiers can reside at a mean pH_T_ 7.7–7.9 at low pH vent sites zones through their life, but are absent at zones with a mean pH ≤ 7.6 (Calosi *et al*., 2013; Kroeker *et al*., 2013; Foo *et al*., 2018). The impact of environmental acidification also varies among regions and habitats with some species or populations appearing to be adapted or phenotypically adjusted to environmental acidification. This is seen in the resilience of sea urchins living in low pH upwelling zones and vent sites (Calosi *et al*., 2013; Hofmann *et al*. 2014; Migliaccio *et al*., 2019). Some molluscs can alter the amount of the form of CaCO_3_ produced (e.g. aragonite or calcite) to a more favourable form making them more resilient to acidification (Fitzer *et al*., 2014a, 2016; Langer *et al*., 2014; Rühl *et al*., 2017).

In calcifying marine invertebrates, environmental carbon for calcification such as dissolved inorganic carbon (DIC) can be sourced in the form of CO_3_^2−^ or hydrogen carbonate (HCO_3_^−^) from ambient seawater. Under CO_2_-driven acidification, this may impair shell growth as pH and CaCO_3_ saturation are lowered resulting in reduced carbonate available for biomineralisation (Doney *et al*., 2009). Therefore, under OA, where CO_3_^2−^ becomes less available from seawater limited shell growth and increased abnormalities become a coping mechanism for continued biomineralisation (Wittmann and Pörtner, 2013). However, respiratory CO_2_ can also be a source of carbon for calcification where it is used to form HCO_3_^−^ through hydrolysis, a process catalysed by carbonic anhydrase (Wilbur, 1972; Roleda *et al*., 2012), a highly conserved enzyme that functions in CO_2_ regulation. Therefore, as DIC sources required for calcification vary between calcifying species, it might also be expected that shell growth responses to OA may be species specific. This is an important consideration in understanding the vulnerability of species and their calcification response in ocean and coastal acidification conditions.

The production and maintenance of CaCO_3_ structures are vital to the success and survival of a vast diversity of marine species as they play essential roles in body support and protection. Corals and oysters provide the structural foundation of reef ecosystems that many species depend on for habitat. Marine calcifiers also provide vital ecosystem services to humanity as species for fisheries and aquaculture and in shoreline protection and are vital to the livelihoods of millions globally (Cooley *et al*., 2009; 2011; Gattuso *et al*., 2015). There has been a wealth of studies on the impacts of OA on marine invertebrates with respect to development, physiology and ecology, as detailed in a number of reviews and meta-analyses (Andersson and Gledhill, 2013; Kroeker *et al*., 2013; Gazeau *et al*., 2013; Byrne *et al*., 2013; Wittmann and Pörtner, 2013; Dubois, 2014; Prezeslawski *et al*., 2015; Foo *et al*., 2018). The mineralogy of marine skeletons and their vulnerability to OA has also been reviewed (Ries, 2010; Smith *et al*., 2013, 2016).

Despite concerns for the prospects for marine calcifiers in a changing ocean, the impacts of environmental acidification on the structure of the biomineral itself remains largely underexplored. We need to understand how acidification alters biomineral production and its microstructure and mechanical integrity to address uncertainties on the biological consequences of climate change. This is addressed here in a review of recent research, where advanced microscopy is used to visualize the internal microstructure and crystallography of biomineral in calcifiers that have been exposed to experimental (laboratory) or natural (vents, coastal run off) acidification (Table 1). Changes in microstructure have been revealed by the application of scanning electron microscopy (SEM) to view surface changes and microcomputed tomography (μCT) to generate three-dimensional reconstructions of entire shells and skeletons (Fitzer *et al*., 2019a).

Several studies have investigated the biomechanical changes to marine skeletons exposed to environmental acidification (Table 1). These studies have largely used crushing tests and nanoindentation. Nanoindentation provides a high-resolution assessment of material hardness and is especially useful on smaller, thinner shell samples using nanoscale indents (depth of 10–100’s nm). In addition, nanoindentation can be used to determine the elastic modulus (Young’s modulus—*E*) of the surface of the shell (Fitzer *et al*., 2015a; Milano *et al*., 2016; Meng *et al*., 2018). Details of the advanced microscopy and materials science techniques that have been used to investigate marine skeletons in an OA context are reviewed in Fitzer *et al*. (2019a).

The list of studies (Table 1) provides an overview of the impacts of environmental acidification on skeletal structure and biomechanics and the pH levels and scales (e.g. pHT vs pHNBS) used are important to note. In the following text, we review research on the major marine calcifying taxa (tube worms, corals, molluscs, echinoderms) with a focus on those most studied, bivalves and sea urchins. Only a few studies have investigated the impact of habitat acidification and warming, and the interaction of these factors, on the structure and integrity of marine biomineral, and we highlight a few of these.

### Corals

Corals produce an aragonite skeleton that is covered by the tissues of the coral polyps (Von Euw *et al*., 2017). Several studies have investigated the impact of OA on the microstructure of coral skeletons using SEM or μCT and one study has incorporated mechanical tests (Table 1). The number of studies appears surprisingly limited considering the ecological importance of reef-building corals. For this important taxon more research is needed on the impact of projected near-future acidification on skeletal structure and mechanical integrity and in context with habitat warming.

The temperate solitary cup coral *Balanophyllia europaea* living at a low pH vent site had a more porous and thinner skeleton than those living at nearby control sites (Fantazzini *et al*., 2015). Skeletal pore size increased and bulk density decreased, with decreasing pH (8.1, 7.9, 7.7). However, growth, with respect to size, was maintained by these corals along the vent pH gradient. Nanoindentation revealed that the skeleton of *B. europaea* living at low pH (pH 7.7) had a lower hardness and stiffness compared with corals residing at pH 8.1. While this species is able to maintain growth at reduced pH, the deposition of less biomineral is likely to increase susceptibility to physical damage (Fantazzini *et al*., 2015).

For colonies of the tropical coral, *Stylophora pistilla*, exposed to pH 7.2 for 12 months, SEM and μCT revealed that the biomineral produced had greater porosity and lower bulk density compared with conspecifics maintained at ambient pH (Table 1). Note the caveat that the low pH treatments used are well beyond OA scenarios (IPCC, 2014) (as the case for many of the studies listed in Table 1). Tambutté *et al*., (2015) suggest that these extreme treatments are useful to help identify trends. While the growth of *Stylophora pistilla*, as linear extension did not change in pH 7.2, the density of the biomineral did, potentially as a trade-off strategy under competition to maintain access to light and space with the compromise of a weaker skeleton (Tambutté *et al*., 2015). This change in the skeleton also reflects the decline in *S. pistilla* colonies at low pH sites in nature (Tambutté *et al*., 2015). A recent study of these species maintained at low pH for 14 months showed a change in skeletal chrystallography (Coronado *et al*., 2019).

Colonies of *Porites astreoides* living in a naturally low pH environment from coastal run off also had a lower density skeleton (Crook *et al*., 2013) and juvenile *Favia fragum* exposed to CO_2_ driven under saturation conditions (pH not provided) for 8 days had a thinner skeleton compared with controls (Cohen *et al*., 2009).

In these coral studies skeletal dissolution was not noted. It appears that the thin veneer of polyp/corallite tissue protects the underlying skeleton from dissolution. Importantly, the most universal measure of coral health and biomineralisation rate (linear extension) is unlikely to be a reliable metric of coral health and growth in the face of OA, with potential production of more fragile phenotypes (Tambutté *et al*., 2015).

### Tube worms

Serpulid polychaetes are a major group of marine worms that produce a calcareous tube that varies in composition from being entirely aragonitic, to entirely high-Mg calcite to mixtures of these two (Smith *et al*., 2013). The serpulid, *Hydroides elegans*, is one of the most important species in tropical biofouling communities and the microstructure and mechanics of its calcareous tube has been investigated in the OA context (Chan *et al*., 2012; Li *et al*., 2014, 2016).

In studies where *H. elegans* was reared in experimental OA conditions across larval development through to tube formation, SEM and μCT revealed that at low pH (7.4–7.8), the tube had increased porosity, decreased thickness and had surface pitting and erosion. There was also a change in the mineral layers and crystallography. These changes reduced the hardness of the tube. The force needed to crush the tube decreased by 62%. The composition of aragonite in the tube is also influenced by OA with *H. elegans* depositing more calcite in the tube at low pH. This species seems to be able to adjust the mineralogy of its tube as a potential adaptation to the changes in water chemistry driven by OA, but this compromises the strength and elasticity of the tube (Li *et al*., 2014, 2016). In comparison, the serpulid, *Spirobranchus triqueter* was similarly fragile when reared under moderate (pH 7.7) and severe (pH 7.4) reductions in pH. Tube fracture toughness was not linearly related to the reduced pH, but was related to changes in porosity as found for *H. elegans*. Larger pores in *S. triqueter* resulted in thinner calcareous layers in the tubes and therefore reduced fracture toughness (Díaz-Castañeda *et al*., 2019).

In experiments where ocean warming was also considered, increased temperature (+6°C) had the opposite effect, increasing skeletal hardness and elasticity (Chan *et al*. 2012). Although the temperature increase used was more extreme than predicted under future climate scenarios, the outcome for this important polychaete indicated that in a future ocean biomineral production may be more dictated by climate warming than acidification.

### Gastropods

Depending on the species and life stage, gastropods produce aragonitic or calcitic shells or a combination of these (Marin *et al*., 2008; Rühl *et al*., 2017). The impact of OA on shell microstructure of several species, maintained in experimental OA for various lengths of time has been investigated (Table 1). For the ecologically important species, *Nucella lapillus* and *Nassarius nitidus*, the reduction in shell density determined using μCT was marked (20–50%), while that for *Columbella rustica* was much less (0.8–8%) (Queirós *et al*., 2015; Chatzinikolaou *et al*., 2017). This shows differences in vulnerability of shells to OA between closely related gastropod species. For the two species greatly affected, the extent of biomineral reduction varied across locations in the shell which would make weaker regions a target for durophagous predators such as crabs. In a study of the mechanical integrity of the shells of *Littorina littorea* maintained in pH 7.8 for 5 months, the shells of larger snails were more vulnerable to the crushing action of a crab predator than those of smaller snails (Landes and Zimmer, 2012). In a study of 7 gastropod species, the mechanical properties of the shells of six of these were not affected by acidification (pH 7.8-7.9) (Leung *et al*., 2017). For *Austrochochlea* constricta, shell hardness and stiffness increased. This species also produced a shell with higher calcite to aragonite and magnesium to calcium ratios under acidification conditions (Leung *et al*., 2017). In this study elevated temperature (+ 2.5-4.0 °C) was the more important factor on shell mechanics. For juvenile *N. lapillus*, grown under OA conditions, a 2°C warming appeared to counter the negative effect of OA, but as this differed between small and large snails, the trend was difficult to interpret (Rühl *et al*., 2017).

Gastropods resident at vents also have thinner, less dense shells as seen in μCT reconstructions (Garilli *et al*. 2015; Harvey *et al*. 2018). The shell surface of *Charonia lampas* exhibited the corrosive effects of low pH water even to the extent that the soft tissue was exposed (Harvey *et al*. 2018). Overall, gastropods living at CO_2_ vent sites tend to have a smaller adult body size, similar to the Lilliput effect seen in the fossil record for mollusc species that survived past low pH driven extinction events (Garilli *et al*., 2015). It is suggested that smaller body size is a physiological adaptation to environmental acidification in order to maintain calcification as well as to have the energy to repair shell dissolution (Garilli *et al*., 2015). For herbivorous gastropods living at vent sites, the enhanced algal food levels due to high CO_2_ can buffer, to a variable extent, the negative effect of OA on calcification (Doubleday *et al*., 2019).

**Figure 2:**
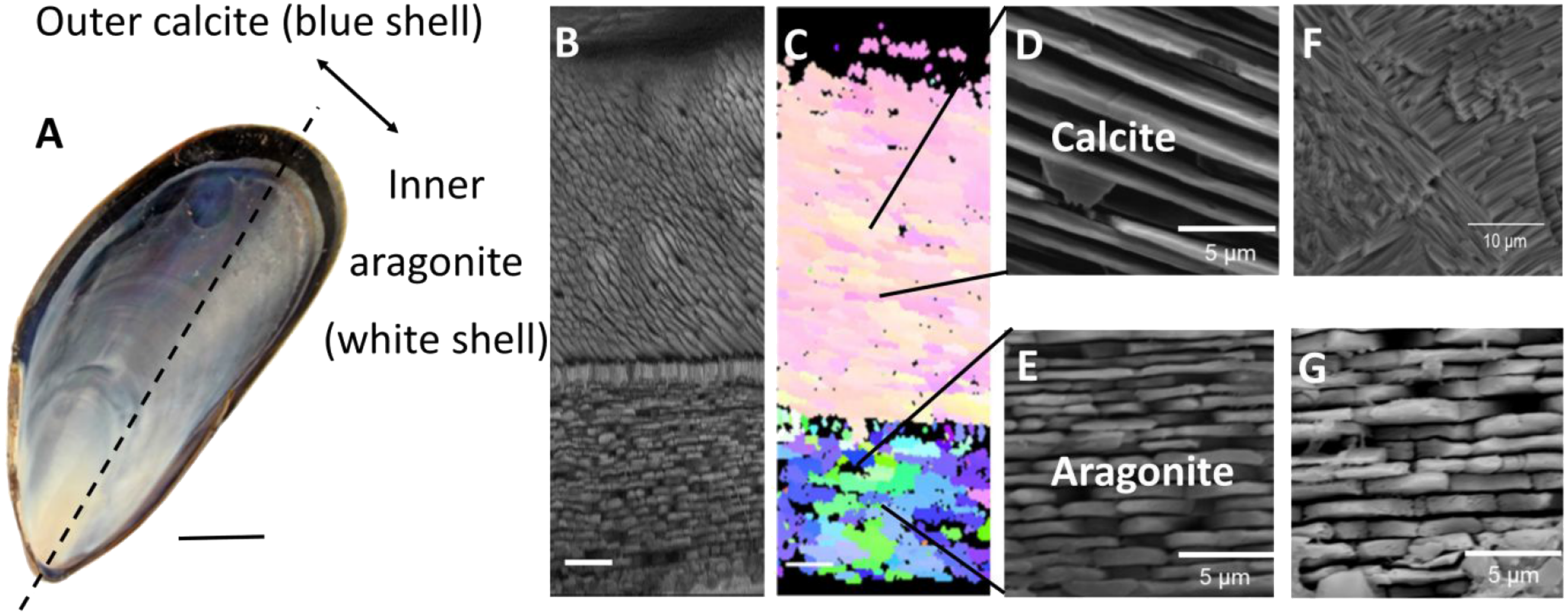
A schematic representation of the use of SEM-EBSD as an analytical tool to assess the effects of acidification on shell microstructure. (**A**) *M. edulis* has been a focal study species to understand the impacts of OA on marine biomineral. (**B**) Section imaged using SEM is a cross section through a shell grown under OA (pHNBS 7.5). (**C**) The same section imaged using EBSD and analysed for crystallographic orientation displayed as a crystallographic orientation map. (**D**) Calcite and (**E**) aragonite crystals at higher magnification from the same cross section of the *M. edulis* shell. (**F**) Disordered microstructure of the calcite layer and (**G**) dissolution of the aragonite tablets (edges more rounded compared to panel E and tablets are less tightly packed) of the shells grown in OA. Images adapted from Fitzer *et al*. (2014a).

### Bivalves

Bivalve skeletons are comprised of two shells joined at a hinge and vary in their composition of calcite and aragonite (Marin *et al*., 2008; Wehrmeister *et al*., 2011). There has been extensive research on the impacts of OA on these animals as they form the basis of significant aquaculture productivity. They are also ecologically important as being major prey for many species and their filter feeding activity strongly influences water clarity and quality.

Many bivalve studies report reduced shell growth, reduced shell thickness and mechanically weaker shells under OA conditions (Gazeau *et al*., 2007; Ries *et al.* 2009; Beniash *et al*., 2010; Dickinson *et al*., 2012; Fitzer *et al*., 2015a, b, 2018). Reduced shell growth and thinning of shells under experimental CO_2_ acidification have been observed using SEM at both the larval (Gazeau *et al*., 2010; Parker *et al*., 2012; Fitzer *et al*., 2014a) and adult stages (Gazeau *et al*., 2013; Fitzer *et al*., 2014b). However, no effect on shape or size of crystals was observed in the clam *Arctica islandica* or the scallop *Adamussium colbecki* under elevated CO_2_ (Stemmer *et al*., 2013, Dell’ Acqua *et al*., 2019). The abnormalities and changes to shell growth can affect bivalves at the microstructure level of the forming phases of aragonite and calcite (Wehrmeister *et al*., 2011), and can also impact the amorphous calcium carbonate (ACC) that is an important precursor of crystalline carbonate minerals (Addadi *et al*. 2003).

Microstructural observations of bivalves exposed to acidification conditions have been determined using a range of microscopy techniques including SEM coupled with electron backscatter diffraction (EBSD) (Melzner *et al*., 2011; Fitzer *et al*., 2014a, b), Fourier-transform infrared (FTIR) spectroscopy (Beniash *et al*., 2010) and X-ray photo emission electron microscopy (XPEEM) (Fitzer *et al*., 2016) to characterize crystallography and the form of calcium carbonate. μCT has been used to examine skeletal density (Meng *et al*., 2018).

In the mussel *Mytilus edulis,* which contains an outer calcite and an inner aragonite layer (Fig. 2), experimental OA resulted in a thinning of the shell (Fitzer *et al*., 2015b), with disordered crystallography as revealed by SEM-EBSD as well as a corroded aragonite layer (Melzner *et al*., 2011; Fitzer *et al*., 2014a), in favour of a disordered calcite layer (Fitzer *et al*., 2014a, b). Examination of the shells of *M. edulis* exposed to CO_2_ acidification with XPEEM revealed that more ACC was produced (Fitzer *et al*., 2016). This was suggested to aid repair to the disordered calcite layer in the shell (Fitzer *et al*., 2016).

**Figure 3:**
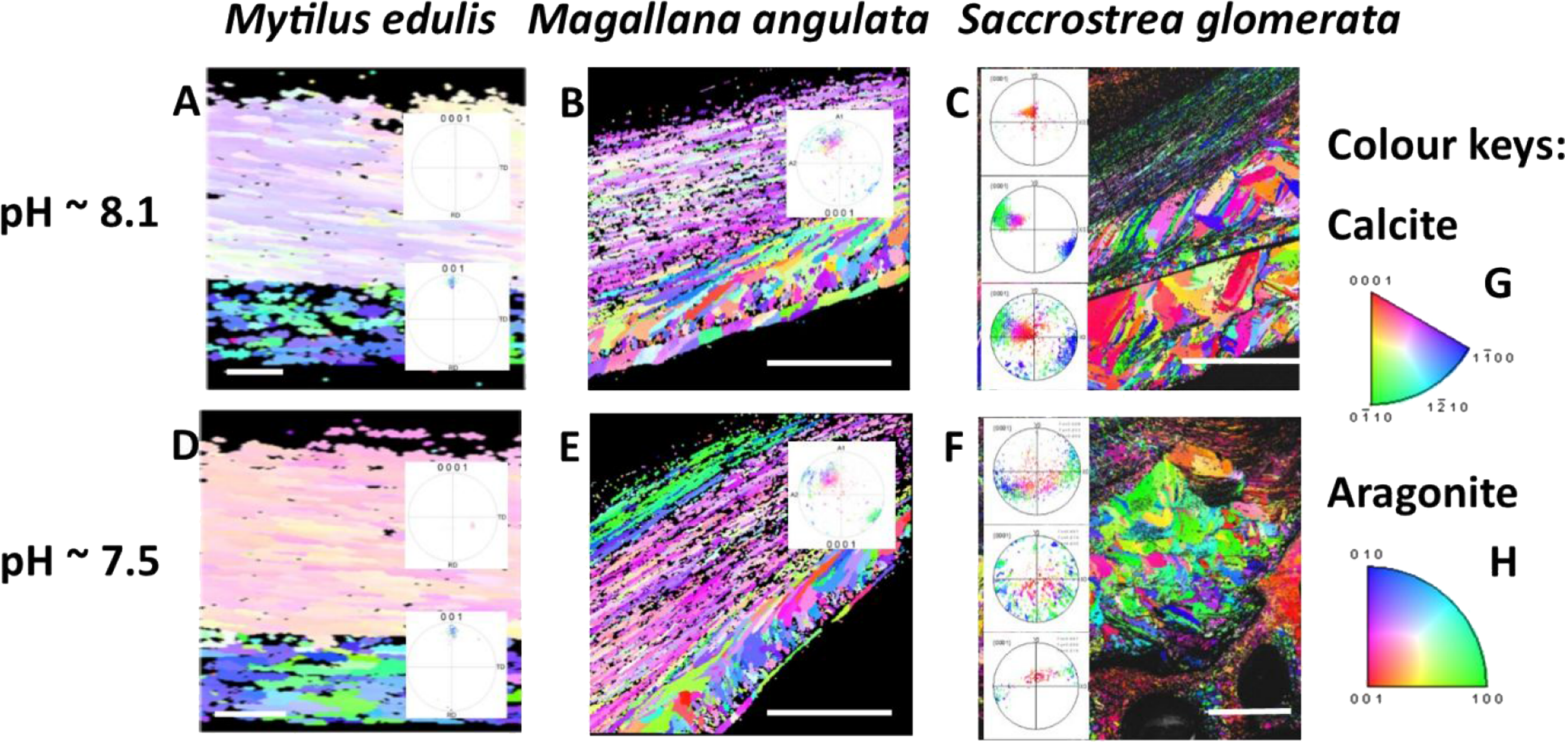
Crystallographic orientation maps with accompanying pole figures for the calcite and aragonite shell of *M. edulis* (**A**, **D**), and the calcite shells of *Magallana angulata* (**B**, **E**) and *Saccostrea glomerata* (**C**, **F**) grown at pH 8.1NBS and pHNBS 7.5 under CO_2_ acidfication and sulphate soil acidification. The figures highlight the similarly altered crystallographic orientation of the mussel and oyster shells with increased disorder at pH 7.5. This is highlighted by the increased range of crystallographic orientation shown by the increased variation of colours. The colours here represent a change in the angle of crystallographic orientation as per the calcite (0001) (**G**) and aragonite (001) (**H**) colour keys. Scale bars represent 5 μm for *M. edulis*, 45 μm for *M. angulata* and 200 μm for *S. glomerata*. Adapted from Fitzer *et al*. (2014a, 2018) and Meng *et al*. (2018).

While *M. edulis* has been a focal case study species (Table 1), many bivalves are similarly affected by OA with commonly observed thinning of the shells and disorder in the crystallography of the calcite layers (Figs 2 and 3). An SEM-EBSD investigation of *Mytilus galloprovincialis* transplanted to a CO_2_ vent (~pH = 7.2–7.8) revealed that they grew a thinner shell with disordered crystallography (Rodolfo-Metalpa *et al*., 2011; Hahn *et al*., 2012). This was also the case the shells of the oysters *Magallana angulata* (pH 7.2, 7.5) and *M. hongkongensis* (pH 7.3) grown in laboratory (CO_2_ dosing) (Meng *et al*., 2018, 2019), and *Saccostrea glomerata* farmed in coastal acidified environments (pH 7.6–7.8) (Fitzer *et al*., 2018, 2019b). The disordered crystallography in *S. glomerata* wild type oysters grown in coastal acidified environments was as a result of altered biomineralisation pathways shown by changing shell carbon isotopes (Fitzer et al., 2019b). Oysters such as the pearl oyster *Pinctada fucata* that have an internal layer of aragonite, dissolution occurred internally under OA conditions (Welladson *et al*., 2011).

The impacts of OA on the biomechanics of bivalve shells have been studied using a variety of techniques, in particular microindentation of the shell surface to calculate hardness. The resultant cracks propagating from the indents can also be applied to calculate fracture toughness (Mackenzie *et al*., 2014; Fitzer *et al*., 2015a). The changes to the shell microstructure in *M. edulis* grown in OA conditions resulted in a mechanically weaker shell as indicated by nanoindentation. The calcite produced was harder and more brittle. Microindentation indicated reduced fracture toughness (Fitzer *et al*., 2015a) and reduced bend/flex before failure (Mackenzie *et al*., 2014).

While OA (pH 7.5) as a single stressor reduced shell hardness and stiffness in *M. edulis*, when this level of acidification is used in combination with moderate warming (+2°C), these negative effects were reduced (Fitzer *et al*., 2015b). Temperature alone did not alter shell size of thickness; however, a combination of +2°C warming with acidification (pH 7.5) reduced the aragonite layer thickness in *M. edulis* (Fitzer *et al*., 2015a). A +4°C warming reduced the maximum crushing load of mussel shells in both ambient and reduced pH (pH 7.6) (Mackenzie *et al*., 2014).

The mechanical properties of bivalve shells that have different forms of CaCO_3_ differ in response to OA. In the shell of *M. edulis*, which contains both calcite and aragonite, the calcite becomes harder or more brittle when exposed to OA. For the calcitic shell of the oyster *Crassostrea virginica* grown under experimental OA, microindentation revealed that shell hardness is reduced along with a resultant reduced fracture toughness (Beniash *et al*., 2010; Dickinson *et al*., 2012). Similarly, the shells of juvenile *Magallana angulata* grown under experimental OA (pH 7.2, 7.5) had reduced hardness, as a result of increased porosity as visualized by μCT (Meng *et al*., 2018). In the more resistant *M. hongkongensis,* juvenile shells were similarly affected, but at a much lower pH (pH 7.3) (Meng *et al*., 2019). For this species, shell hardness and crystallography remained unchanged at pH 7.6 (Meng *et al*., 2019). Oysters such as *Pinctada fucata* that have an internal layer of aragonite had a weaker shell when grown under experimental OA conditions (Welladsen *et al* 2011) than oysters that have a calcitic internal layer. The shells of *S. glomerata* growing in estuarine habitats with sulphate soil acidification (pH 6.6, 6.8, 6.9) were also significantly weaker in crushing tests (Amaral *et al*., 2012). Although these pH levels are much lower than predicted OA, this level of acidification occurs in acid sulphate regions during increased rainfall (Amaral *et al*., 2012). Reduced growth of *S. glomerata* during such extreme events may be offset by positive growth in dry periods (Amaral *et al*., 2012).

Overall, OA affects the microstructure of bivalve shells through reduced shell growth with thinner, more porous shells that have reduced fracture toughness. Trends appear similar in mussels and oysters in terms of microstructural responses to OA regardless of whether laboratory CO_2_ acidfication or environmental acidification from CO_2_ or coastal run off are the source of acidification (Table 1).

### Echinoderms

**Figure 4:**
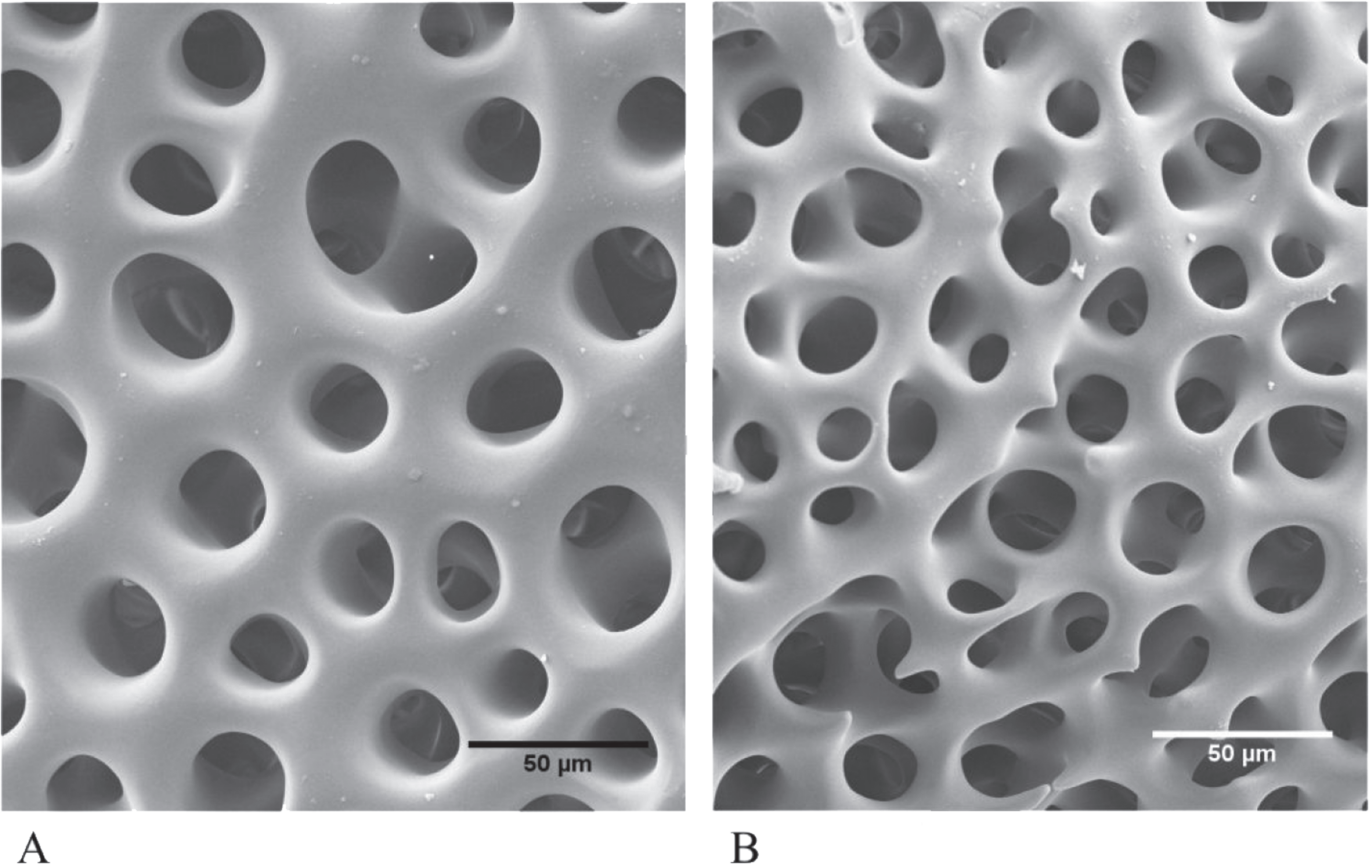
SEM of the surface of the apical test plates of the adult sea urchin, *Heliocidaris erythrogramma* maintained in control (pHNBS 8.1) (**A**) and decreased pH (pHNBS 7.6) (**B**) for 9 months. The skeleton formed in the OA treatment has thinner calcite. Images courtesy of Ms R. Johnson.

Echinoderms produce a unique endoskeleton laid down as a three-dimensional mesh-like calcite lattice (Fig. 4), with cells and connective tissue living in the void space (Cavey and Markel, 1994). The impacts of OA on the production and maintenance of the skeleton are extensively investigated for echinoids (sea urchins) across their planktonic and benthic life phases (Byrne *et al*. 2013; Dubois, 2014). These animals are ecologically and economically important grazers across world oceans (Lawrence, 2013). The sea urchin skeleton is composed of Mg-calcite, ~3–16 wt% MgCO_3_, where Mg^2+^ is substituted for Ca^2+^ during calcification (Chave, 1954; Smith *et al*., 2016). As a result of this chemical composition, the echinoderm skeleton is one of the most soluble forms of CaCO_3,_ and so is vulnerable to dissolution in OA conditions (Andersson *et al*., 2008; McClintock *et al*., 2011; Dubois, 2014).

Despite the echinoderm skeleton being a form that is vulnerable to dissolution, the epithelium largely protects the living skeleton from direct exposure to low pH conditions (Dubois, 2014). The exceptions are the tips of the spines, potentially due to their thin epithelial cover. Overall, the established sea urchin skeleton does not appear to be vulnerable to direct dissolution under OA conditions (Table 1). Surface pitting of the test has only been reported at very low pH (Holtmann *et al*., 2013). Interesting, the cidaroids, a group of sea urchins with many species living in deep water below the isocline, produce spines that do not have an epithelial cover, but have a calcitic cortical layer that makes them resistant to dissolution in OA conditions, as shown for 15 species, including in tests where species were maintained in pH 7.2–7.4 for 3–5 weeks (Dery *et al*., 2014, 2017, 2018).

**Figure 5:**
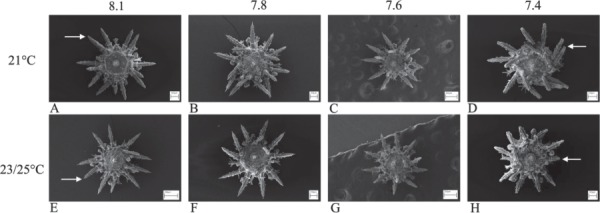
SEM of juvenile *Heliocidaris erythrogramma* reared in four pH and three temperature levels in all combinations for 14 days. Urchins had shorter spines and smaller tests at pHNBS 7.4 (see Wolfe et al. 2013b). At control pH (**A**, **E**) the arrows point to the terminal spike which is the calcite growing region of the spines compared with the flat-ended spines of juveniles reared in pHNBS 7.4 indicating retarded or no calcification. Images courtesy of Dr K Wolfe.

**Figure 6:**
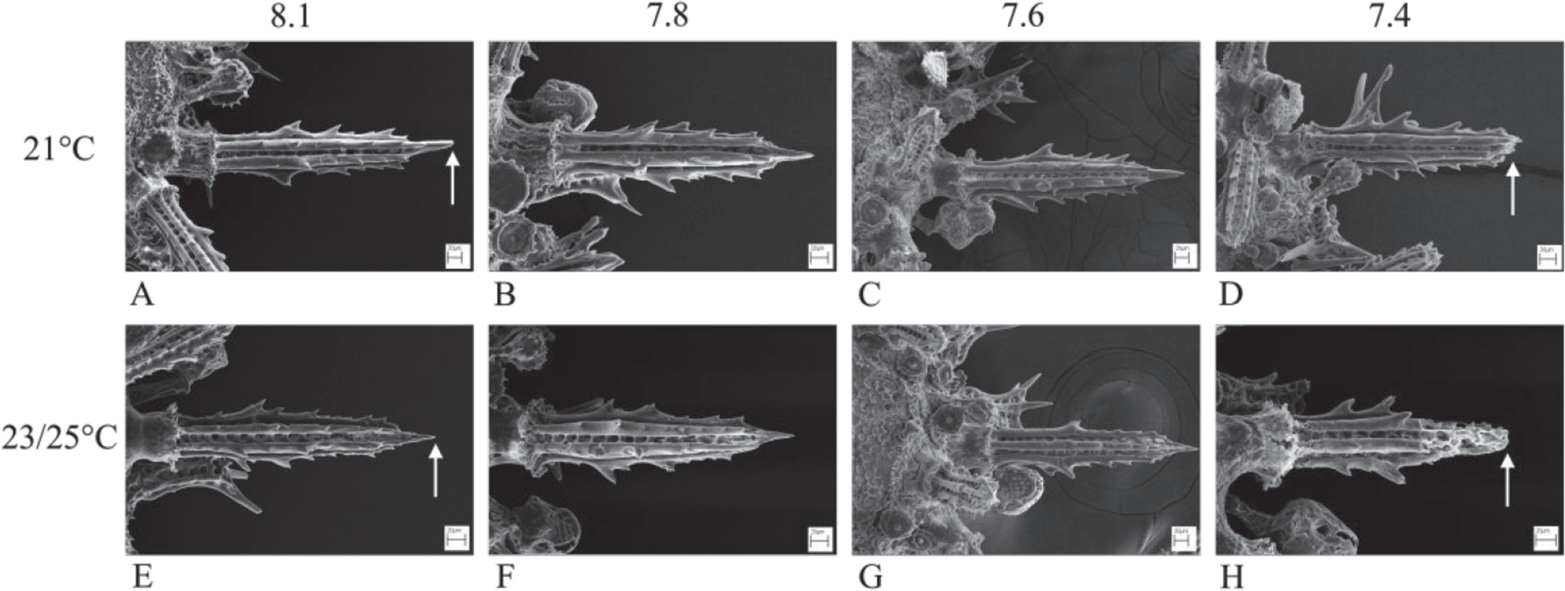
SEM of the spines of juvenile *Heliocidaris erythrogramma* reared in four pH and three temperature levels in all combinations for 14 days. The arrows point to the pointed end of the spines in control pHNBS 8.1 (**A**, **E**) at the calcite growing region. At pHNBS 7.4 and at warmer temperature the spines were shorter, more porous, had blunt ends (**D**) and were eroded (**H**) (see Wolfe et al. 2013b). Images courtesy of Dr K Wolfe.

**Figure 7:**
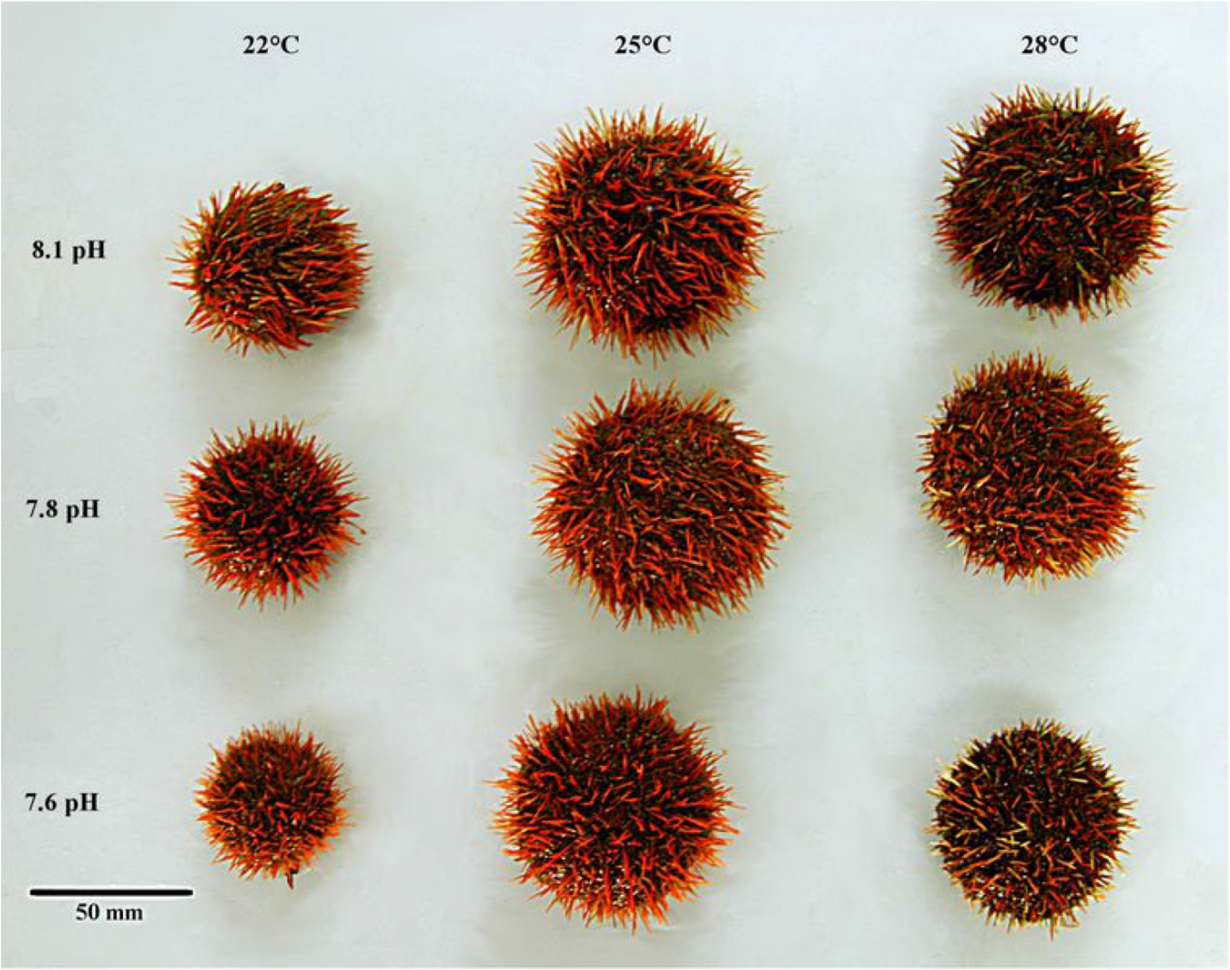
*Tripneustes gratilla* reared in three pH and three temperature levels in all combinations from the early juvenile (5.0 mm test diameter) for 146 days. A +3°C warming mitigated the negative effects of low pHNBS (pH 7.6, 7.8), but further warming was deleterious. From Dworjanyn and Byrne (2018).

With respect to production of biomaterial, calcification in sea urchins is constrained by OA. This is shown by the comparatively smaller skeletons of larvae and juveniles (Figs 5 and 6) reared in OA conditions through development and the smaller tests of adult sea urchins (Fig. 7) grown in long-term OA experiments (Byrne *et al*., 2013, 2014; Dubois, 2014; Dworjanyn and Byrne, 2018). It appears that sea urchins have a reduced ability to deposit biomineral under OA conditions (Figs 4 and 5). This may be due to energetic constraints associated with the higher metabolic costs of life at low pH and the priority to maintain acid-base balance (Stumpp *et al*., 2012; Carey *et al*., 2016). The impacts of OA on the skeleton may be mitigated by near future warming. In *Tripneustes gratilla*, a +3°C warming mitigated the negative effects of low pH (pH 7.6, 7.8) on test growth (Fig. 7). Further warming made matters worse (Byrne *et al*., 2014; Dworjanyn and Byrne, 2018).

In addition to the smaller amount of skeleton produced under OA conditions, skeletal microstructure may also be altered. Several SEM and/or μCT investigations have compared the pore size, the calcitic trabecular connections and the void space of the skeleton of sea urchins exposed to OA with these traits in the skeleton formed under ambient pH (Table 1). Larger pore size is reported for the spines of juvenile *Heliocidaris erythrogramma* (Fig. 6), but only at very low pH (pH 7.4) (Wolfe *et al*., 2013b) and for the teeth of *Paracentrotus lividus* where the tips were regenerated under OA (pH 7.7) (Asnaghi *et al*., 2013).

For *H. erythrogramma* maintained on OA conditions for 9 months, the mid body ambital test plates produced prior to exposure to OA exhibited no change in skeletal structure. In contrast, the younger apical plates from the active growth region of the test, that had likely grown over the 9-month exposure at low pH had thinner trabeculae and a greater void space compared to those from control urchins as visualized with SEM (Fig. 4A and B) and μCT (Johnson, 2019).

The μCT study of *Strongylocentrotus fragilis* that had likely grown from the juvenile stage in chronic low pH (pH 7.69–7.57) and oxygen saturation on the Californian continental shelf, showed that that skeletal porosity and pore size is higher than that for conspecifics living at higher pH. For this species, skeletal porosity increased with decreasing pH and oxygen saturation, but it is not known how hypoxia and low pH interact with respect to the differences in the skeleton (Sato *et al*., 2018).

The impact of OA on the mechanical properties of the test plates has been documented by nanoindentation and in crushing and bending tests (Table 1). In *P. lividus* maintained in OA (pH 7.8, 7.9) for 12 months, there was no effect of pH on the hardness or breaking force of the test plates (Collard *et al*., 2016), as also the case for *Echinometra mathaei* (Moulin *et al*., 2014). Exposure to low pH reduced the hardness of the apical plates of *H. erythrogramma* that had likely been produced in OA conditions, but the previously established ambital plates were not affected (Johnson and Byrne, unpublished data). There was also no change in the mechanical properties of the test of *P. lividus* resident at a vent site (~pH 7.7) compared with conspecifics from ambient conditions.

In studies where the whole test was crushed as might occur in an attack by a predatory fish (Guidetti and Mori, 2005), the force required to crush the dried tests of juvenile *P. lividus* and *Diadema africanum* maintained in OA was lower compared to juveniles from control treatments (Asnaghi *et al*., 2013; Rodríguez *et al*. 2017). However, as dried tests were used, the ecological relevance of these results is not clear. The force required to crush the test was lower in live *Tripneustes gratilla* reared in OA (pH 7.6) from juvenile to adult in a test designed to mimic the ram force of a predatory fish, but the break occurred at the suture lines, not in the skeleton itself (Byrne *et al*., 2014). These tests need to be repeated across species with live urchins being most relevant test subjects, to incorporate the elasticity of the ligaments that bind the test plates (Ellers *et al*., 1998), and with application of forces modelled with respect to the jaw crushing or ramming action of predators, although these forces are poorly characterized.

With respect to the spines of sea urchins maintained at pH 7.4 and 7.7, the thinner, more brittle spines formed in these conditions by *Tripneustes ventricosus* and *Eucidaris tribuloides* are weaker in bending tests, but not the spines of *Prionocidaris baculosa* (Dery *et al*., 2017). While the spines are the most vulnerable skeletal element in sea urchins to OA, these structures are designed to break in a predatory attack, and regenerate quickly (Dery *et al*., 2017). But this entails an energetic cost (Haga *et al*., 2016)

Overall, OA affects the material properties of the sea urchin skeleton through changes in growth rate with less biomineral produced. While it appears that there is weakening of the sea urchin skeleton in response to OA, thereby compromising its protective roles, more studies are needed where characterisation of the impacts of OA on biomineral microstructure is investigated in tandem with biomechanical tests and in skeletal elements that have been completely produced at low pH. Finally, the 3D void space of the sea urchin endoskeleton is important for the resident cells and tissues. Any change in the stereotypic arrangement of the skeleton is likely to have impacts for organism function beyond the skeleton itself.

### Crustaceans

The crustacean exoskeleton is made of chitin and calcite and is comparatively tolerant of acidification due to the lower degree of calcification (Whiteley, 2011), the exception being adult barnacles, with several studies on these animals (Table 1). For *Amphibalanus amphitrite*, there were no effects of low pH (to pH 7.4) through the larval stage, but the adult shell was weaker (McDonald *et al*., 2009). Corrosion of the shell of *A. improvises* was evident at low pH (Pansch *et al*., 2014). Increased temperature (+4°C) resulted in an increase in shell strength of this species, but there was no effect of pH (Pansch et al. 2013, 2014). For *Balanus improvises*, low pH (stable pH 7.7, fluctuating pH 7.5–7.9) reduced the strength of the shell (Eriander *et al*., 2016).

## Discussion

The morphological and biomechanical properties of marine invertebrate skeletons are integral to animal function and individual fitness which in turn affects population dynamics and community structure (Kroeker *et al.,* 2014). In a climate change world, it is important to understand how calcification systems, and the biomineral that is produced, respond to environmental acidification. The combination of detailed morphology using advanced imaging techniques and biomechanics, is emerging as a leading-edge approach to quantify the vulnerability of marine skeletons. This approach is also key to detecting the potential for phenotypic adjustment in biomineralisation as a means to produce and maintain biomineral in the face of environmental acidification.

Although the field of marine climate change has focused on anthropogenic CO_2_-driven acidification, many important calcifying species live in shallow coastal habitats that are vulnerable to acidification from increasing run off due to pressures from sea level rise and precipitation (Amaral *et al*., 2011; Fitzer *et al*., 2018). The chemistry of ‘ocean’ and ‘coastal’ acidification, and how they impact the carbonate system, the basis for calcification, are fundamentally different (Fig. 1). For bivalves, despite these differences, the resulting morphology and biomechanics of the biomineral produced under the two forms of acidification are similar (Beniash *et al*., 2010; Rodolfo-Metalpa *et al*., 2011; Dickinson *et al*., 2012; Hahn *et al*., 2012; Fitzer *et al*., 2018; Meng *et al*., 2018). Coastal waters have a more variable chemistry than the ocean and understanding how this chemistry interacts with CO_2_-driven acidification, remains a significant challenge. This is especially important for socioeconomically important mollusc resources that are typically fished and cultured in coastal bays and estuaries.

In response to environmental acidification, the biomineral produced by marine invertebrates across many taxa, is more porous and less dense. Increased skeletal porosity in diverse species has been revealed by SEM and/or high-resolution 3D reconstructions generated by μCT. Indeed, some skeletal malformations are only evident through application of these methods (Fitzer *et al*., 2014a, b, 2015a, b, 2018; Rühl *et al*., 2017). There may be a balance between the absolute amount of mineral that can be physiologically deposited in the skeleton in order to maintain its key physiological functions and the energy that can be devoted to this function under OA. For molluscs and echinoderms, constraints in biomineral production result in smaller body size, similar to the Lilliput effect seen in the fossil record when smaller mollusc species survived global warming extinction events (Garilli *et al.,* 2015). In contrast for corals, growth, as measured by linear extension of the skeleton, the standard metric of coral health appears to be maintained under CO_2_-driven acidification, but the skeleton produced has a lower density and is thus more vulnerable to physical damage (Tambutté *et al*., 2015). OA also impacts biomineral directly through dissolution and this is most commonly noted for molluscs that lack an outer protective cover of conchiolin (Ries *et al*., 2009; Rodolfo-Metalpa *et al*., 2011; Harvey *et al*., 2018). Echinoderm skeletons are protected from corrosion by their epithelial cover, and in corals, the polyp tissue protects the skeleton.

Within each of the groups considered here, environmental acidification impacts skeletal microstructure and biomechanics in different ways, reflecting the diverse calcification biology of the species investigated. In bivalves, where skeletal CaCO_3_ varies in composition of calcite and aragonite, the responses to CO_2_-driven acidification are often similar within the same family. For example, the mytellids (*Mytilus* sp) have harder, brittle shells prone to fracture under OA, compared with the Ostreidae (*Crassostrea* sp.) where the shells have reduced hardness. Despite these different responses, the outcome with regard to the mechanical integrity of the shell of mussels and oysters is similar with reduced fracture toughness of shells formed in OA conditions.

Volcanic CO_2_ vent systems have been used as natural laboratories to investigate the impacts of near and far future OA (Foo *et al*., 2018; González-Delago and Hernández, 2018). The CO_2_ gradient at these sites provides a pH range from very low ~pH 6 to ambient levels in nearby habitats not affected by CO_2_ venting. Investigation of marine calcifiers resident along these CO_2_ gradients where all or most of their skeleton has formed under low pH, has been an important means to understand how acidification impacts calcification. Overall, the results of laboratory and vent studies, at similar pH/acidification levels, are similar with respect to the response of marine invertebrate calcification. For herbivorous species resident at vents, higher levels of algal resources promoted by the CO_2_ helps to ameliorate the energetic stress caused by acidification (Uthicke *et al*., 2016).

Biomechanical tests of the biomineral produced under experimental and natural acidification indicate that the shells and skeletons of many species are weaker than those produced in ambient conditions. This has been largely shown for molluscs with some studies of tube worms and corals, although the shells of many gastropod species are not affected by acidification (Leung et al., 2017). For echinoderms, biomechanics tests indicated that the spines are most affected by acidification (Collard *et al.*, 2016; Dery *et al.*, 2017) and that the newly formed apical plates were weaker (Johnson and Byrne, unpublished data). Overall, the shift to producing weaker skeletons with increased porosity decreased density under acidification conditions in many species will compromise the function of these structures in protection against physical stress and defence against predators.

It is key to consider the fitness consequences of observed change. Change to animal size, strength or defence will have effects to marine communities beyond the fitness of the individual. Changes to predator–prey interactions may change predation rates of vulnerable species and thereby alter population dynamics and community structure in the future (Kroeker *et al*., 2014). Weaker shells change predator–prey dynamics as durophagous predators such as crabs optimize their foraging to preferentially target weaker-shelled individuals or weaker regions of the shell of their molluscan prey (Kroeker *et al*., 2014). Predatory mollusc and boring epibionts also target the weaker, less dense areas of shells to drill and bore through the biomineral. Many molluscs also exhibit an inducible defence behaviour producing stronger, more calcified shells in response to the presence of a predator or in response to hydrodynamic conditions and the latter also applies to sea urchins (Bibby *et al*., 2007; Collard *et al*., 2016). For the gastropod *Littorina obtusata*, the inducible defence response expressed in the presence of a crab predator was inhibited at low pH, albeit at a rather extreme level (pH 6.6, ~ 14 000 μatm *p*CO_2_) (Bibby *et al*., 2007). The thinner shells of the oyster *Ostrea lurida* reared from the larval stage in OA appears to have made them more vulnerable to predatory gastropods (Sanford *et al*., 2014). In a study of *M. edulis* and the drilling gastropod *Nucella lapillus* maintained in the same acidification conditions, the thinner mussel shells produced in OA conditions made them more vulnerable to predation by the gastropod (Sadler *et al*., 2018). The outcomes for marine calcifiers in the face of OA will also be influenced by other environmental factors such as food levels. Mussels and barnacles are able to maintain a higher-level calcification in the presence of high food levels (Melzner *et al*., 2011; Pansch *et al*., 2014). Sea urchins with a higher level of calcified algae in their diet produce stronger skeletons (Asanaghi *et al*., 2013). Species adapted to fluctuations in intertidal habitats are also more tolerant of acidification (Wolfe et al., 2013b; Leung et al., 2017).

For several marine invertebrate groups across a variety of biomineralized structures, the lack of studies of the microstructure and mechanics stands out as gaps to address. In particular, the response of the crustacean chitin-calcite exoskeleton to acidification is poorly studied. The impact of acidification on jellyfish calcium sulfate (basanite) statoliths (Mooney and Kingsford, 2016; Sötje *et al*., 2017) is not known. Understanding the effects of acidification on the organic matrix of marine skeletons is also an important gap in knowledge, especially as shell matrix proteins are essential for crystal nucleation and other key aspects of skeletogenesis (Marin *et al*., 2008).

Finally, while the integrated morphology-mechanics approach has great potential to characterize the impacts of environmental acidification on marine invertebrate skeletons and advance our understanding of the biological consequences of climate change, this approach has only been applied to a relatively small number of species (Table 1). There is great interest in how calcification and the biomineral produced will be altered in a changing ocean and it is clear that application of morphology and ecomechanics, in context with use of realistic pH levels, will be particularly informative.
